# Efficacy of adjunctive photodynamic therapy to conventional mechanical debridement for peri-implant mucositis

**DOI:** 10.1186/s12903-024-04198-6

**Published:** 2024-04-16

**Authors:** Jincai Guo, Xueru Chen, Hui Xie, Tongjun Li

**Affiliations:** 1 Changsha Stomatological Hospital, No. 389 Youyi road, Tianxin district Changsha, Changsha, Hunan 410006 China; 2 School of Stomatology, Hunan University of Chinese Medicine, Changsha, 410006 China

**Keywords:** Peri-implant mucositis, Photodynamic therapy, Mechanical debridement, Meta-analysis

## Abstract

**Objective:**

This meta-analysis was conducted to assess the effectiveness of photodynamic therapy (PDT) as an adjunct to conventional mechanical debridement (CMD) for the management of peri-implant mucositis (p-iM).

**Methods:**

We systematically searched four databases (PubMed, Embase, Web of Science, and Cochrane Library) for randomized controlled trials (RCTs) investigating PDT + CMD for p-iM from their inception to March 13, 2023. Meta-analysis was performed using RevMan 5.4 software.

**Results:**

Seven RCTs met the inclusion criteria. The meta-analysis revealed that PDT + CMD treatment was more effective than CMD alone in reducing probing depth (PD) (Mean Difference [MD]: -1.09, 95% Confidence Interval [CI]: -1.99 to -0.2, *P* = 0.02) and plaque index (PI) (MD: -2.06, 95% CI: -2.81 to -1.31, *P* < 0.00001). However, there was no statistically significant difference in the improvement of bleeding on probing (BOP) between the PDT + CMD groups and CMD groups (MD: -0.97, 95% CI: -2.81 to 0.88, *P* = 0.31).

**Conclusions:**

Based on the current available evidence, this meta-analysis indicates that the addition of PDT to CMD significantly improves PD and PI compared to CMD alone in the treatment of p-iM. However, there is no significant difference in improving BOP.

**Supplementary Information:**

The online version contains supplementary material available at 10.1186/s12903-024-04198-6.

## Introduction

Peri-implant mucositis (p-iM) denotes inflammatory changes in the mucosal tissues surrounding dental implants, characterized by inflammation occurring in the absence of any loss of underlying bone support. This condition is often attributed to plaque-induced inflammation affecting both the peri-implant and palatal soft tissues [1]. Assessment of inflammation includes parameters such as bleeding on probing (BOP), erythema, swelling, and, in some cases, suppuration may manifest as well [2, 3]. P-iM is a prevalent issue in patients with dental implant restorations, with an estimated prevalence of approximately 20% among individuals who do not undergo regular periodontal maintenance therapy [4], This figure rises to around 50% among noncompliant patients [5]. The formation of bacterial biofilms on implant surfaces has been identified as a contributing factor to p-iM’s etiology. Irregular bacterial biofilms on implant surfaces can compromise implant osseointegration and induce inflammation in the surrounding mucosal tissues [6]. Furthermore, the influence of other risk factors, such as smoking, a history of periodontal disease, and diabetes, should not be underestimated in this multifaceted process [7, 8]. P-iM is a reversible condition, it can lead to oral discomfort, pain, swelling, and other symptoms that affect the patient’s quality of life and oral health. However, if not treated in time, it can lead to serious consequences, such as the spread of infection and implant failure. These can cause psychological and physical harm to patients, increase the economic burden on patients and their families, and increase the medical burden on society. Consequently, various treatment modalities for p-iM have been developed and evaluated [9]. In clinical practice, mechanical debridement is considered the “gold standard” for managing peri-implant diseases [10], with adjunctive therapies like laser therapy (LT), antimicrobial agents, antibiotics, and photodynamic therapy (PDT) also proving effective [11].

PDT, an acronym for photodynamic therapy, represents a non-invasive phototherapy modality wherein a light source interacts with photosensitizers (PSs), inducing light toxicity that leads to cellular damage and death [12]. PDT finds application in the treatment of various medical conditions, including acne, psoriasis, age-related macular degeneration, herpes infections, cancer, and various oral diseases [13–15]. While several randomized controlled trials (RCTs) have demonstrated the effectiveness of PDT in addressing p-iM, there exist certain controversies surrounding its efficacy for this condition. Some studies have reported the efficacy of PDT in effectively treating p-iM [16–20], while others have indicated that PDT has no significant impact on bleeding and plaque index associated with p-iM [21]. Hence, a comprehensive meta-analysis is warranted to assess the role of PDT as an adjunct to conventional mechanical debridement (CMD) in managing p-iM. The objective of this meta-analysis is to offer valuable clinical insights into p-iM by systematically evaluating existing clinical RCTs that have investigated the role of PDT in its treatment.

## Methods

### PICO question

The PICO (Participants, Intervention, Control, and Outcomes) question for this study can be framed as follows: “In patients with p-iM, does the addition of PDT to CMD result in more effective treatment outcomes compared to CMD alone?” In this context, P represents patients with peri-implant mucositis, I represents PDT, C represents CMD, and O stands for the improvement of p-iM symptoms, including parameters such as PD, BOP, and PI.

### Information sources and search strategy

The protocol for this meta-analysis was prospectively registered with PROSPERO [22] under the code CRD42023427417. Our search strategy involved a combination of free text terms and Medical Subject Headings (MeSH terms) derived from the PICO framework. We conducted comprehensive searches in four major English-language databases: PubMed, Embase, Cochrane Library, and Web of Science, covering the period from their inception up to March 3, 2023. We specifically targeted RCTs related to the treatment of p-iM using PDT in conjunction with CMD. Additionally, we manually reviewed the reference lists of the included articles in this review. The search strategy was structured as follows:

- #1: (MeSH Terms) Mucositis OR (MeSH Terms) Periimplantitis.
- #2: Title/Abstract Keywords: Periimplant Disease, Peri-implant Disease, Peri-implant Infection, Periimplant Infection, Peri-implant Mucositis, Periimplant Mucositis, Peri-implantitis.
- #3 #1 OR #2.
- #4 (photodynamic therapy [Title/Abstract]).
- #5 #3 AND #4.

### Eligibility criteria

Inclusion Criteria: (1) Study Type: RCTs. (2) Study Subjects: Individuals diagnosed with p-iM through pathological diagnosis or clinical manifestations, irrespective of their race or gender. (3) Intervention Measures: The experimental group employed PDT in conjunction with CMD, while the control group solely utilized CMD. (4) Outcomes: Assessment of PD, BOP, and PI.

Exclusion Criteria: (1) Cases of p-iM comorbid with systemic diseases or other oral mucosal conditions. (2) Studies with ambiguous criteria for inclusion. (3) Data that is either incomplete or erroneous. (4) Articles that lack full-text or abstract availability. (5) Investigations where both experimental and control groups received PDT treatment.

### Study selection and data extraction

In the initial screening phase, articles were excluded based on title and abstract content. During the subsequent thorough screening stage, the full texts of potential articles were scrutinized. Following full-text assessment, selected articles were included based on a predefined data extraction template. Guo J and Chen X independently screened the literature and extracted data, and in cases of discrepancies, Xie H and Li T provided input for resolution. The particulars of each study were extracted, encompassing the primary author’s name, publication year, baseline characteristics, such as age, gender, sample size, specific interventions, risk of bias assessment, and pertinent treatment outcomes of the study subjects.

### Quality assessment

Two reviewers (Guo J and Chen X) independently evaluated the risk of bias. Risk of bias was assessed using the RCT risk assessment tool recommended by the Cochrane Manual 5.1.0.

### Statistical analysis

Statistical analysis was conducted using RevMan 5.4 software. Continuous data were assessed through the calculation of the mean difference (MD) and corresponding 95% confidence interval (CI). Heterogeneity was evaluated employing the chi-square test (α = 0.1) and the inconsistency index statistic (I^2^). In cases where no heterogeneity was observed (*P* > 0.1, I^2^ ≤ 50%), fixed-effects modeling was employed. Conversely, when heterogeneity was present (*P* ≤ 0.1, I^2^ > 50%), we conducted further analysis to identify the sources of significant clinical heterogeneity. Subsequently, a random-effects model was utilised for meta-analysis.

## Results

### Literature search

**Fig. 1:**
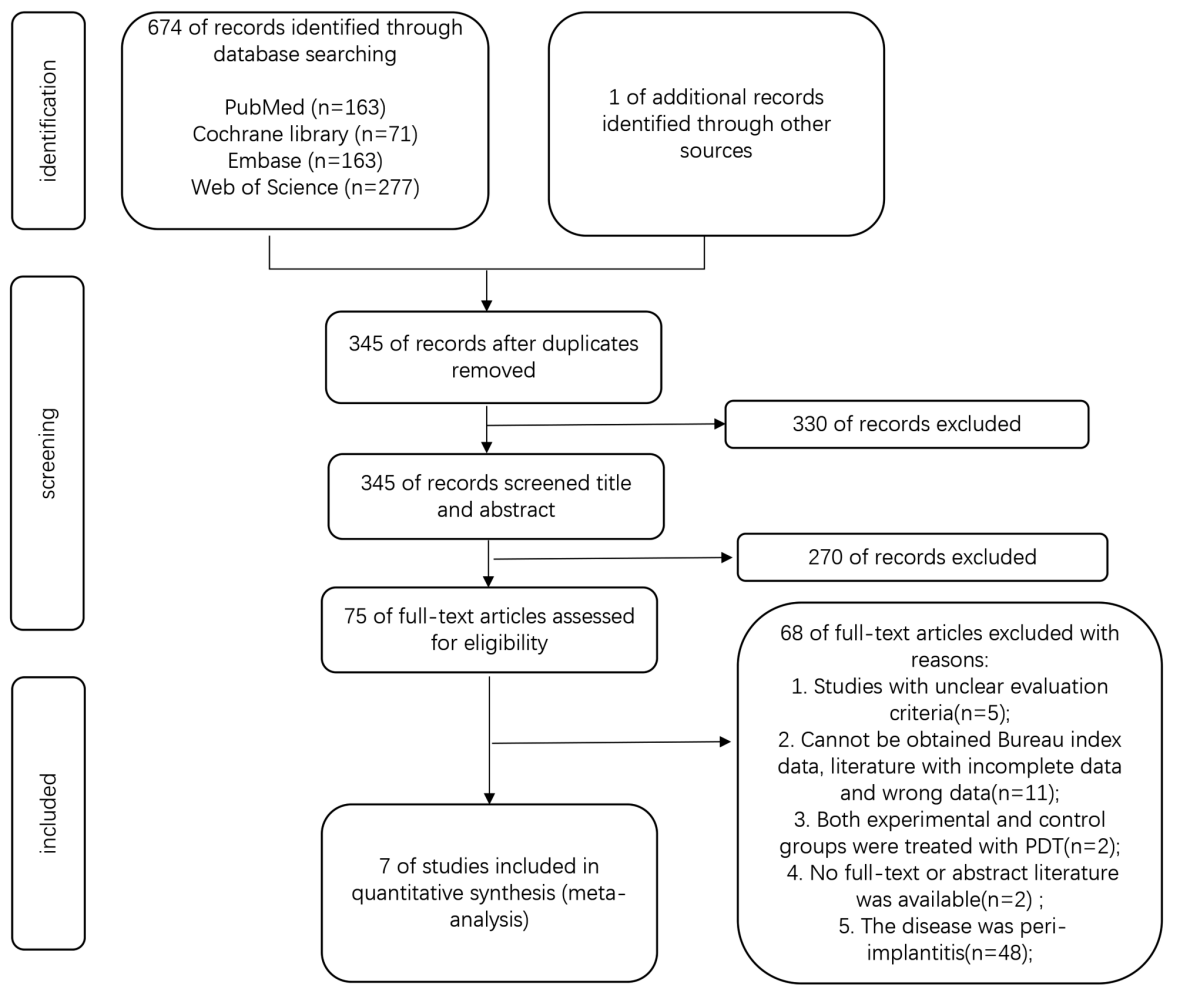
Flow chart of the study selection

A total of 674 relevant studies were initially identified. Additionally, one article was sourced through a manual examination of the reference lists of other articles. After excluding 328 duplicate studies, the titles and abstracts of the remaining 74 articles were screened. Upon full-text assessment, 67 publications were subsequently excluded. The detailed screening process is illustrated in Fig. 1. Based on the predefined criteria, seven RCTs were deemed eligible for inclusion in the meta-analysis.

### Study quality evaluation

The meta-analysis comprised seven studies, all of which were RCTs. Among these, two studies employed specific random sequence generation methods, including computer-generated randomization tables, coin tossing, and online randomizers. The remaining studies did not specify the method used for randomization. Two studies mentioned allocation concealment through the use of sealed opaque envelopes. All included studies provided complete data and did not selectively report any information. The evaluation of study quality is presented in Table 1.

**Table 1:** Quality assessment of included randomized controlled trials

The first author, the year	Random Sequence Generation	Allocation concealment	Blinding of Participants and Personnel	Blinding of Outcome assessment	Incomplete outcome data	Selective reporting	Other sources of bias
Pourabbas,2023 [21]	computer-generated	unclear	doubleblinded	unclear	no	no	unclear
Aldosari, 2023 [17]	unclear	unclear	doubleblinded	unclear	no	no	unclear
Javed, 2017 [20]	tossing a coin	unclear	unclear	unclear	no	no	unclear
Alsayed, 2023 [18]	Online randomizer	Sealed nontransparent envelopes	unclear	unclear	no	no	unclear
Deeb, 2020 [19]	unclear	unclear	doubleblinded	unclear	no	no	unclear
Shetty, 2022 [23]	computer-generated	unclear	doubleblinded	unclear	no	no	unclear
Al Rifaiy, 2018 [16]	tossing a coin	Sealed nontransparent envelopes	doubleblinded	unclear	no	no	unclear

### General characteristics and clinical parameters

The characteristics of the included studies encompassed the first author’s name, publication year, and baseline sample characteristics, which included sample size, gender distribution, and age. These studies, published between 2017 and 2023, involved a total of 295 participants, with 150 allocated to the PDT groups and 145 to the control groups. CMD in the control groups was performed using either sterile hand curettes or titanium curettes. In contrast, the PDT + CMD groups underwent laser exposure for either 10 s [19, 20] or 60s [16–18, 21, 23] after the introduction of various PSs into the pockets surrounding each implant via a blunt needle. Follow-up periods ranged from 3 months to 12 weeks. Among the included studies, two used indocyanine green as the PS [18, 21], two employed phenothiazine chloride [19, 20], and three utilised methylene blue [16, 18, 23]. The primary outcome measures included PD, BOP, and PI. General characteristics and clinical parameters of the included RCTs are summarised in Table 2. The main results and conclusions is presented in Table 3.

**Table 2:** Characteristics of included studies

The first author, year	Participants T C		Gender (M/F) T C		Age (Yr) T C		Intervention T C		Duration	Outcomes	Photosensitizer	Wavelength	Time of irradiation
Pourabbas, 2023 [21]	26	26	/	/	26‒58	26‒58	aPDT + CMD	CMD	3 months	①②	A	805 nm	60s
Aldosari, 2023 [17]	24	23	14/10	12/11	50.3 ± 6.7	52.1 ± 5.1	aPDT + CMD	CMD	12 weeks	①②③	/	660 nm	60s
Javed,2017 [20]	28	26	28/0	26/0	50.6 ± 0.8	52.2 ± 0.5	aPDT + CMD	CMD	12 weeks	①②③	B	660 nm	10s
Alsayed, 2023 [18]	20	20	13/7	10/10	56. ± 6.6	57.5 ± 4.1	PDT + CMD	CMD	3 months	①②③	A and C	810 nm	60s
Deeb, 2020 [19]	15	15	15/0	15/0	52.6 ± 0.9	49.2 ± 0.13	aPDT + CMD	CMD	12 weeks	①②③	B	660 nm	10s
Shetty, 2022 [23]	17	17	/	/	42.5 ± 6.4	45.1 ± 3.3	aPDT + CMD	CMD	3 months	①③	C	660 nm	60s
Al Rifaiy, 2018 [16]	20	18	20/0	18/0	33.6 ± 2.8	35.4 ± 2.1	aPDT + CMD	CMD	12 weeks	①②③	C	670 nm	60s

Outcomes:①probing depth (PD)②bleeding on probing (BOP)③plaque index (PI)

A: Indocyanine green B: Phenothiazine chloride C: Methylene blue

**Table 3:** Main results and conclusions

	PD		BOP		PI		Conclusions
The first author, the year	Experimental	Control	Experimental	Control	Experimental	Control	Conclusions
Pourabbas, 2023 [21]	-1.88 ± 0.8	-1.5 ± 1.25	-27.52 ± 23.41	-45.67 ± 20.3	/	/	The addition of PDT to mechanical therapy did not provide any additional improvements in the clinical or biological parameters of peri‑implant mucosal inflammation.
Aldosari, 2023 [17]	-4.66 ± 0.7	-3.2 ± 0.2	-3.3 ± 0.05	-0.98 ± 0.04	-2.6 ± 0.2	-1.1 ± 0.07	One session of aPDT after MD with adjunct aPDT is effective in reducing soft tissue inflammation in patients with PiM.
Javed, 2017 [20]	-5.9 ± 0.3	-2.8 ± 0.4	-1.4 ± 1.1	-1.7 ± 0.7	-37.2 ± 9.2	-28 ± 5.7	MD with adjunct aPDT is more effective in the treatment of peri-implant mucositis in smokers compared with MD alone.
Alsayed, 2023 [18]	-0.68 ± 0.75	-0.84 ± 0.76	-27.78 ± 26	-27.66 ± 26.6	-28.94 ± 28.2	-24.15 ± 29	PDT showed statistically significant improvements in peri‑implant clinical, radiographic, microbiological, and immunological parameters as compared to conventional MD.
Deeb, 2020 [19]	-0.9 ± 1.1	-0.4 ± 0.9	-4.3 ± 4.4	-1.8 ± 4	-33 ± 8.4	-30.5 ± 7.1	PDT as an adjunct to MD is as efficacious as adjunctive AB therapy. However, additional benefits in the reduction of bleeding scores were observed for PDT in peri-implant inflammation among cigarette smokers.
Shetty, 2022 [23]	-4.2 ± 0.2	-1.9 ± 0.28	/	/	-2.3 ± 0.4	-0.8 ± 0.2	A single session of aPDT as an adjunct to MD is effective in reducing peri-implant soft tissue inflammation and OYC in patients with PIM.
Al Rifaiy, 2018 [16]	-2.2 ± 0.7	-2.3 ± 0.8	-2.9 ± 2.9	-1.3 ± 0.9	-37.9 ± 9.2	-19.3 ± 8.4	Antimicrobial PDT is more effective compared to MD alone in the treatment of p-iM in individuals vaping e-cigs.

PD: probing depth BOP: bleeding on probing PI: plaque index

### Study outcomes

#### Probing depth

**Fig. 2:**
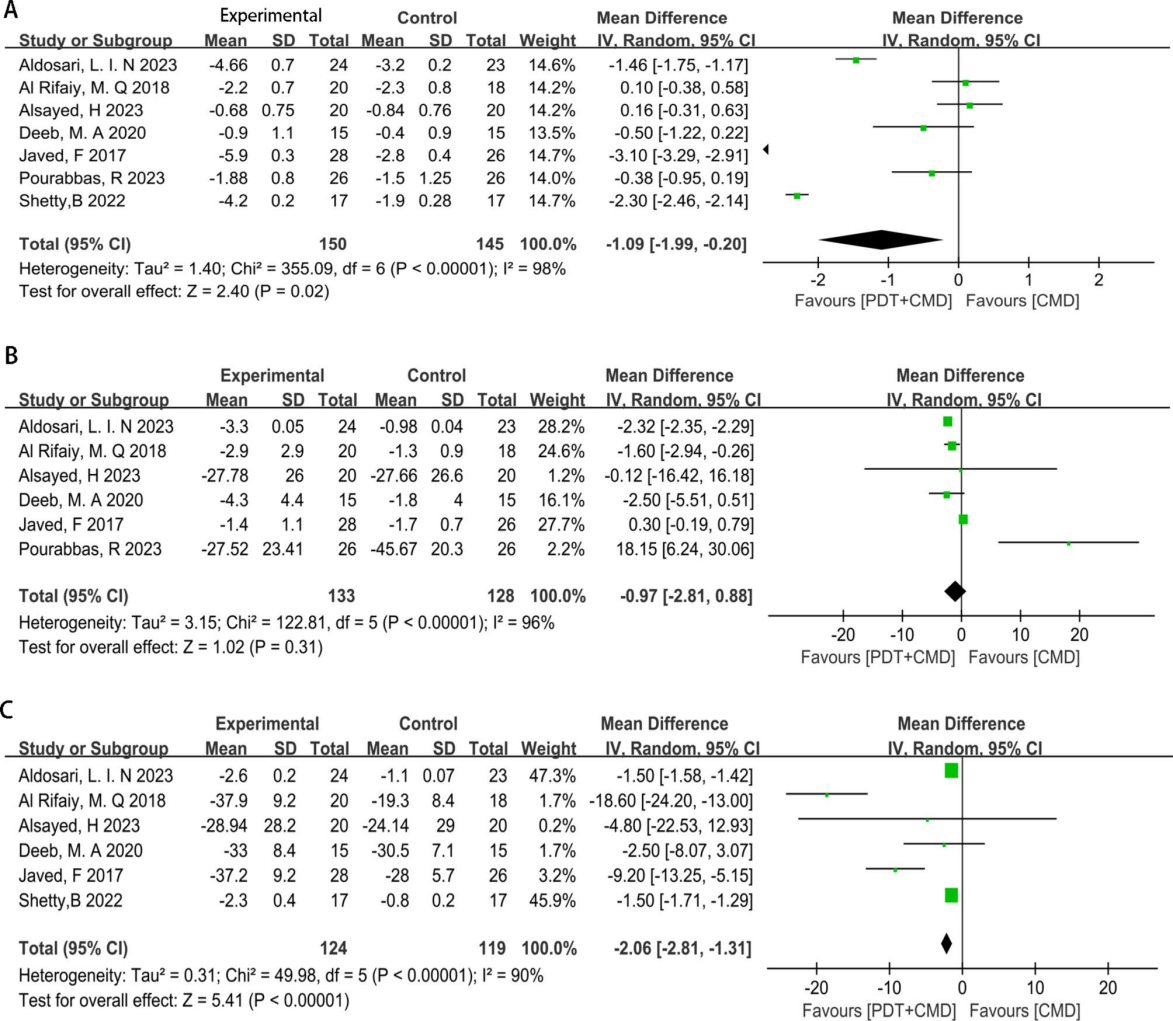
Meta-analysis for the treatment effects between the PDT + CMD and CMD group. (A) PDT + CMD is more effective in the improvement of probing depth. (B) There is no significant difference in the improvement of bleeding on probing. (C) PDT + CMD is more effective in the improvement of plaque index

All studies [16–21, 23] incorporated PD as an outcome measure. The combined data, as depicted in Fig. 2A, indicated that PDT + CMD treatment outperformed CMD in enhancing PD (MD: -1.09, 95% CI: -1.99 to -0.2, *p* = 0.02, I^2^ = 98%).

### Bleeding on probing

**Fig. 3:**
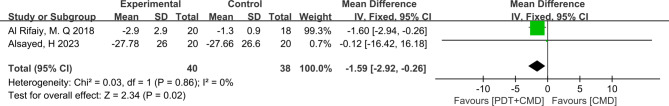
Meta-analysis of indocyanine green as a photosensitizer in the improvement of BOP between PDT + CMD and CMD

**Fig. 4:**
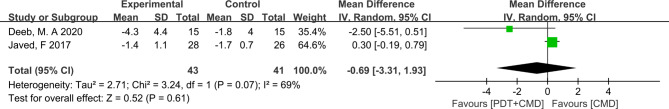
Meta-analysis of phenothiazine chloride as a photosensitizer in the improvement of BOP between PDT + CMD and CMD

Six studies [16–21, 23] evaluated BOP. As illustrated in Fig. 2B, the results revealed no statistically significant difference in BOP improvement between the PDT + CMD groups and CMD groups (MD: -0.97, 95% CI: -2.81 to 0.88, *p* = 0.31, I^2^ = 96%). Given the variation in the PSs used across the studies, we conducted a subgroup analysis to explore potential differences in treatment effects based on PS type. The subgroup analysis, categorised by the PS used in the PDT + CMD groups, is presented in Figs. 3 and 4. It was found that PDT + CMD treatment yielded more favorable BOP improvement when methylene blue was employed as the PS (MD: -1.59, 95% CI: -2.92 to -0.26, *p* = 0.02, I^2^ = 0%). However, no significant difference was observed in BOP improvement between the PDT + CMD groups and CMD groups when phenothiazine chloride was used as the PS (MD: -0.69, 95% CI: -3.31 to 1.93, *p* = 0.61, I^2^ = 69%).

### Plaque index

Six studies examined the PI [16–20]. As presented in Fig. 2C, the pooled data from these studies demonstrated that PDT + CMD treatment was more effective than CMD in reducing PI (MD: -2.06, 95% CI: -2.81 to -1.31, *p* < 0.00001, I^2^ = 90%).

## Discussion

Pi-M is a common complication following dental implant. CMD is considered as the gold standard for treating pi-M. However, CMD is often unable to completely remove the bacterial biofilm, and there are some limitations. In clinical practice, adjunctive treatments, such as laser therapy (LT), antibacterial agents, antibiotics, and PDT, are commonly used to improve treatment outcomes for pi-M. Among them, the adjunctive use of PDT for pi-M has attracted the attention of researchers due to its promising therapeutic efficacy.

PDT represents a distinctive treatment modality involving the use of PS and harmless light sources [24]. When the PS is exposed to this benign light, it becomes activated and generates cytotoxic oxygen species, such as singlet oxygen or free radicals. This process leads to membrane disruption, targeted cell destruction, and protein inactivation [25–27]. Importantly, PDT does not result in scarring post-treatment and reduces the risk of recurrence [28], rendering it a highly promising therapeutic approach. While PDT has been explored as a treatment for p-iM [29], studies have confirmed its efficacy in this context [16–21, 23, 30–33]. However, there is still controversy regarding the effectiveness of PDT in improving certain indicators for pi-M patients due to variations in study populations, duration of irradiation, and the use of PSs. Therefore, conducting a systematic meta-analysis is necessary.

The primary question addressed in this meta-analysis is: “Is PDT adjunctive CMD more effective than CMD alone when used to treat p-iM??” Our meta-analysis data revealed that PDT + CMD treatment was superior to CMD alone in enhancing PI and PD. However, no significant difference was observed in improving BOP. A meta-analysis conducted by Shahmohammadi, R et al. [33] also demonstrated that antimicrobial PDT (aPDT) significantly improved PI and PD compared to mechanical debridement alone in smokers with peri-implantitis or p-iM. Additionally, a study by Al-Sowygh et al. [31] indicated that mechanical debridement in conjunction with aPDT was more effective in reducing inflammation in smokeless tobacco product users with p-iM compared to mechanical debridement alone. These findings are closely related to our meta-analysis results, indicating that adjunctive use of PDT is indeed effective in the treatment of peri-implant diseases, regardless of whether the patients are smokers or non-smokers, or whether they are peri-implantitis or pi-M.

Our meta-analysis results show that PDT significantly reduces PI. One in vitro study investigated the effect of low-level laser therapy (LLLT) and PDT on bacterial count, and the results showed that PDT was more effective in reducing bacterial count [34]. Another systematic review concluded that PDT could reduce the number of bacteria around dental implants [35]. The main mechanism of improving PI is that PSs can release free oxygen or free radicals to effectively combat bacteria without harming surrounding tissues under light irradiation, thereby improving PI.

PD is also known as the periodontal pocket depth, one of the symptoms of pi-M is an increase in PD [36]. Our meta-analysis results show that PDT can significantly improve PD. Krane et al. [37] found that matrix metalloproteinases (MMPs) were upregulated in periodontitis and peri-implant inflammation. MMPs can degrade collagen fibers, the increased expression of MMPs can lead to tissue destruction around dental implants. Javed et al. [38] reported that the levels of tumor necrosis factor (TNF-α), interleukin (IL)-6, IL-1β, and other inflammatory cytokines in peri-implantitis were increased. This suggests that these cytokines may also affect the development of pi-M. A previous study showed that adjunctive PDT could lead to a decrease in destructive inflammatory cytokines (such as TNF-α, IL-1β, MMP-8, and MMP-9) in gingival crevicular fluid, promoting wound healing [39]. The mechanism of improving PD and supporting wound healing is that PDT can reduce destructive inflammatory cytokines and MMPs, heighten collagen synthesis, and increase cell proliferation [40].

PDT showed no significant difference in improving BOP, given the variability in outcomes, we conducted a subgroup analysis that revealed differential effects based on the use of PSs. PSs are chemical compounds that, when exposed to light energy, undergo reactions in the presence of molecular oxygen, resulting in the production of cytotoxic agents such as singlet oxygen (_1_O^2^) or superoxide (O^2−^), ultimately inducing cellular damage [41, 42]. Consequently, PSs are pivotal components in the implementation of PDT. PSs encompass three broad categories: (1) porphyrin-based PSs; (2) chlorophyll-based PSs; and (3) dyes. In our meta-analysis, dye-based PSs were employed. Methylene blue classified as a phenothiazine dye, can be administered topically or orally and is recognized for its non-toxic properties. Its outstanding photochemical characteristics render it the preferred choice for addressing superficial oral lesions [43, 44]. Consequently, methylene blue emerges as an excellent PS for treating pi-M. Our subgroup analysis further underscored that PDT in conjunction with CMD significantly enhances the mitigation of BOP when methylene blue serves as the PS.

Some studies [16, 17, 19, 20] included in our analysis used antimicrobial PDT, which is a common treatment. However, antibiotics usually are associated with side effects, including antibiotic resistance and dysbacteriosis [45]. The inappropriate use of traditional antibiotics in dental practice has led to an increase in antibiotic resistance. Recent studies [46, 47] have shown that antimicrobial peptides (AMPs) are candidates as an alternative to conventional antibiotic treatment for oral diseases caused by bacteria. They can lyse bacterial cells by interacting with the cell membrane. In the future, CMD, PDT, and other interventions in conjunction with AMPs may provide better therapeutic effects in combating dysbiosis and preventing the onset and progression of oral infections.

It is noteworthy that PDT is exceptionally well-tolerated and safe, with no reported adverse reactions in the literature included in our analysis. Our meta-analysis has some advantages and innovations. Firstly, we have obtained reliable results through a reasonable study design and comprehensive literature search. Secondly, compared with previous study [48], we have included a wider range of populations, not limited to smokers or diabetics, with a larger number of participants. Finally, we draw an objective conclusion that PDT is beneficial in improving PD and PI in patients with p-iM, which provides a reference for clinical management.

Nonetheless, our analysis is not without limitations. Firstly, the number of included studies was limited, and the sample sizes were relatively small. Secondly, the populations included in these studies were inconsistent, with some focusing exclusively on p-iM patients who smoked, while others did not specify smoking status. Finally, the literature we included exhibited variations in PDT parameters. There was no consensus regarding laser wavelength, application frequency, or the use of different PSs across the literature, potentially impacting the overall effectiveness of PDT.

## Conclusion

In conclusion, this meta-analysis highlights the potential of adjunctive PDT alongside CMD in significantly improving PD and PI when compared to CMD alone in the treatment of p-iM. However, it’s important to note that no significant difference was observed in BOP. Given the limitations of small sample sizes in the included RCTs and the substantial heterogeneity in evaluation indicators, further RCTs featuring larger sample sizes, multicenter settings, and extended follow-up durations are warranted to establish more definitive conclusions.

### Electronic supplementary material

Below is the link to the electronic supplementary material.

Supplementary Material 1

Supplementary Material 2

Supplementary Material 3

## Data Availability

The datasets used and/or analysed during the current study are available from the corresponding author on reasonable request.
