# Supplementary material for: Efficacy of adjunctive photodynamic therapy to conventional mechanical debridement for peri-implant mucositis
Source: BMC Oral Health. 2024 Apr 16;24:464. doi: 10.1186/s12903-024-04198-6 (PMC11020816; doi:10.1186/s12903-024-04198-6)
Supplement: Supplementary file 2 — Supplementary Material 2 [file 12903_2024_4198_MOESM2_ESM.docx]

**Identification of studies via databases and registers**

Records removed *before screening*:

Duplicate records removed (n =309)

Records marked as ineligible by automation tools (n = 0)

Records removed for other reasons (n = 20)

Records identified from*:

Databases (n =675)

Registers (n = 0)

**Identification**

Records screened

(n = 345)

Records excluded**

(n = 270)

Reports sought for retrieval

(n =75)

Reports not retrieved

(n = 68)

**Screening**

Reports excluded:

1. Studies with unclear evaluation criteria(n=5);

2. Cannot be obtained Bureau index data, literature with incomplete data and wrong data(n=11);

3. Both experimental and control groups were treated with PDT(n=2);

4. No full-text or abstract literature was available(n=2);

5. The disease was peri-implantitis(n=48);

Reports assessed for eligibility

(n = 7)

Studies included in review

(n = 7)

Reports of included studies

(n = 0)

**Included**

*Consider, if feasible to do so, reporting the number of records identified from each database or register searched (rather than the total number across all databases/registers).

**If automation tools were used, indicate how many records were excluded by a human and how many were excluded by automation tools.

*From:*  Page MJ, McKenzie JE, Bossuyt PM, Boutron I, Hoffmann TC, Mulrow CD, et al. The PRISMA 2020 statement: an updated guideline for reporting systematic reviews. BMJ 2021;372:n71. doi: 10.1136/bmj.n71

For more information, visit: <http://www.prisma-statement.org/>
