# Supplementary material for: Efficacy of adjunctive photodynamic therapy to conventional mechanical debridement for peri-implant mucositis
Source: BMC Oral Health. 2024 Apr 16;24:464. doi: 10.1186/s12903-024-04198-6 (PMC11020816; doi:10.1186/s12903-024-04198-6)
Supplement: Supplementary file 3 — Supplementary Material 3 [file 12903_2024_4198_MOESM3_ESM.docx]

**PubMed**

| #1 | **(mucositis[MeSH Terms]) OR (Periimplantitis[MeSH Terms])** | 3773 |
| --- | --- | --- |
| #2 | **((((((periimplant disease[Title/Abstract]) OR (peri-implant disease[Title/Abstract])) OR (peri-implant infection[Title/Abstract])) OR (periimplant infection[Title/Abstract])) OR (peri-implant mucositis[Title/Abstract])) OR (periimplant mucositis[Title/Abstract])) OR (peri-implantitis[Title/Abstract])** | 3839 |
| #3 | #1 OR #2 | 5885 |
| #4 | **(Photodynamic Therapy[Title/Abstract])** | 26712 |
| #5 | #3 AND #4 | 163 |

**Embase**

| #1 | **'periimplant disease'**:ab,ti OR **'peri-implant disease'**:ab,ti OR **'peri-implant infection'**:ab,ti OR **'periimplant infection'**:ab,ti OR **'peri-implant mucositis'**:ab,ti OR **'periimplant mucositis'**:ab,ti OR **'peri implantitis'**:ab,ti OR **mucositis**:ab,ti OR **periimplantitis**:ab,ti | 22732 |
| --- | --- | --- |
| #2 | 'photodynamic therapy':ab,ti | 29576 |
| #3 | #1 AND #2 | 163 |

**Cochrane library**

| #1 | MeSH descriptor: [**mucositis**] explode all trees | 435 |
| --- | --- | --- |
| #2 | (**periimplant disease** s):ti,ab,kw OR (**peri-implant disease**):ti,ab,kw OR (**peri-implant infection**):ti,ab,kw OR (**periimplant infection**):ti,ab,kw OR (**peri-implant mucositis**):ti,ab,kw OR (**periimplant mucositis**):ti,ab,kw OR (**peri implantitis**):ti,ab,kw | 1862 |
| #3 | #1 or #2 | 2220 |
| #4 | **Photodynamic Therap**:ti,ab,kw | 2723 |
| #5 | #3 and #4 | 72 |

**Web Of Science**

| #1 | TS=( **mucositis** ) OR AB=( periimplant disease OR peri-implant disease OR peri-implant infection OR periimplant infection OR peri-implant mucositis OR periimplant mucositis OR peri-implantitis ) | 25227 |
| --- | --- | --- |
| #2 | AB=( **Photodynamic Therapy** ) | 35236 |
| #3 | ( TS=( mucositis ) OR AB=( periimplant disease OR peri-implant disease OR peri-implant infection OR periimplant infection OR peri-implant mucositis OR periimplant mucositis OR peri-implantitis )) AND (TS=( Photodynamic therapy )) | 277 |
